# Unemployment, health, and education of HIV-infected males in Germany

**DOI:** 10.1007/s00038-015-0750-3

**Published:** 2015-10-01

**Authors:** Mona Groß, Annika Herr, Martin Hower, Alexander Kuhlmann, Jörg Mahlich, Matthias Stoll

**Affiliations:** Düsseldorf Institute for Competition Economics (DICE), Heinrich-Heine University of Düsseldorf, Düsseldorf, Germany; CINCH, Universität Duisburg-Essen, Essen, Germany; Klinikum Dortmund, Dortmund, Germany; Centre for Health Economics Research, University of Hanover, Hanover, Germany; Janssen KK, Tokyo, Japan; Centre for Internal Medicine, Hannover Medical School (MHH), Hanover, Germany

**Keywords:** AIDS/HIV, Unemployment, Antiretroviral therapy, Frailty, Job loss

## Abstract

**Objectives:**

The present study on people living with HIV/AIDS (PLWHA) identifies socio-demographic and health-related factors corresponding with their labour market participation.

**Methods:**

The study sample bases on a German observational sub-study of 527 male PLWHA. The present analysis is restricted to male PLWHA in working age. By means of a multivariate regression, we identify factors that contribute to unemployment and job loss.

**Results:**

The probability to be unemployed is significantly negatively correlated with age above 40 years and graduation from university and positively correlated with problems with daily activities (frailty) and disease severity (CDC stage C). The probability of employment loss during the 2-year observation period is significantly negatively correlated with the educational level, whereas frailty and hepatitis C (HCV) co-infection increase the odds of employment loss.

**Conclusions:**

As problems to manage daily activities and disease progression are associated with unemployment, an effective HIV treatment is an important cornerstone for employment. This is also true for the management of comorbidities, such as HCV co-infection, which also negatively affects employment status in our study.

## Introduction

The human immune deficiency virus (HIV) causes a chronic infection, leading to progressed loss of immunological defensive forces (so called immunosenescence) (Deeks 2011) and premature ageing (frailty) (Erlandson et al. 2013) which may finally progress to the acquired immune deficiency syndrome (AIDS). It is characterised by certain life-threatening opportunistic infections and malignancies. Since combined antiretroviral therapy (cART) became broadly available in 1996, disease progression can be effectively delayed by reducing both mortality and morbidity associated with HIV infection (Pichenot et al. 2012). In addition, the effective reduction of viral load as a result of cART substantially reduces the risk of HIV transmission (Chen et al. 2012).

Furthermore, cART improves life conditions of people living with HIV/AIDS (PLWHA) (Goldman and Bao 2004). As a consequence, seeking “normality” appears to be a major aspect of self-perception of PLWHA (Mühlbacher et al. 2013). While the life expectancy of timely antiretroviral treated HIV-infected adults converges to that of the general population, there are still imbalances with regard to the labour market (Worthington et al. 2012; Dray-Spira et al. 2008). Thus, unemployment rates among PLWHA ranging from 45 to 65 % (Dray-Spira et al. 2006) indicate that the working reality of PLWHA is still far from normality.

Several studies have identified relevant factors which may help to explain higher unemployment rates and a higher risk of employment loss of PLWHA (Worthington et al. 2012). The results of these studies demonstrate that unemployment of PLWHA is associated with socio-demographic and occupational factors in addition to health characteristics. The main socio-demographic risk factors identified for unemployed PLWHA include younger age or a poor educational level (Dray-Spira et al. 2006, 2007a, b, 2008). Relevant health-related risk factors comprise the advanced stage of the HIV disease (Dray-Spira et al. 2007a), the time of diagnosis before the launch of cART in 1996 (Dray-Spira et al. 2007a), hepatitis C co-infection (Richardson et al. 2010; March et al. 2007) and psychiatric comorbidities such as depression (Rabkin et al. 2004).

As symptoms and consequences of chronic diseases such as HIV infection occur individually and in different combinations, earlier studies identified parameters that represent impairments of daily life activities in PLWHA (Anandan et al. 2006). Consequently, patient reported problems to manage daily life activities were used here to reflect health-related physical impairments, which we subsume as frailty in the following.

Previous results show that the loss of employment appears in 46 % of the affected persons within 1 year after HIV infection (Dray-Spira et al. 2006), which indicates that the risk of becoming unemployed is highest shortly after infection. Furthermore, those studies also show that women as well as patients with an impaired socio-economic status, progressed HIV infections or concomitant diseases bear a higher risk of becoming unemployed.

To our knowledge, the most recent study on the relationship between unemployment and HIV infection is based on French data of 2002 (Dray-Spira et al. 2006, 2007a, 2008). As the medical innovation this field is progressing at fast pace we see the need to a have a fresh look into this matter to validate or augment previous findings. Moreover, there has been no study on Germany so far. As Germany and France significantly differ in terms of the institutional settings of their labour markets, we might observe different findings for Germany. Unlike France, Germany has undergone painful reforms in the early 2000s to make the labour market more flexible for unskilled workers. This policy has apparently resulted in a significant drop of the unemployment rate (Krebs and Scheffel 2013).

The estimated prevalence of PLWHA in Germany as of 2013 is 1 per thousand, with 81 % of those infected being male. More than 3000 annual incident cases are estimated for 2013 and 2014. The most common transmission risks are sexual contact between men (MSM: 66 % of prevalent cases and 67 % of incident cases), heterosexual contacts (prevalence: 23 %, incidence: 17 %) and intravenous drug use (prevalence: 10 %, incidence: 9 %). An estimated 17 % of PLWHA have an undiagnosed HIV infection. More than 80 % of the diagnosed cases receive antiretroviral therapy (RKI 2014). On these grounds, this German study at hand identifies socio-demographic as well as health-related self-reported (anamnestic) factors corresponding (1) with employed versus unemployed PLWHA and (2) with PLWHA staying in employment vs. becoming non-employed during the 2-year observational period.

## Methods

Data used here stem from the Cost and Resource Utilisation Study in Antiretroviral Therapy (CORSAR), a nationwide German multicentre, non-interventional, prospective cohort study to evaluate economic aspects of HIV treatment using modern cART (Kuhlmann et al. 2015).

A total of 1162 PLWHA representing a 2.3 % sample of treated PLWHA in Germany (RKI 2013a, b) were observed and all health- and work-related issues were documented over 96 weeks between April 2009 (first patient started) and April 2012 (last patient finished) with scheduled quarterly visits to the involved physician. Before that, all participants from the eight practices specialised in HIV and the four hospitals with HIV as a main area distributed across Germany went through a 6-month period with baseline examinations. The study design comprised that diagnostic regimes had not been pre-defined but were purely based on the physician’s decisions. Cost-based information and information on health status stem from the practice or hospital documentation while information on employment status was also quarterly asked for in a patient’s questionnaire.

Inclusion criteria consist of confirmed HIV diagnosis, age of at least 18 years, cART at study entry and written informed consent. The baseline data documentation included age, gender, date of HIV diagnosis, HIV disease staging, treatment line and previous treatment substances. At every scheduled visit, clinical routine data such as viral load, CD4 cell count or blood pressure and concomitant diseases were recorded. Additionally, all visits at physicians, hospitalisations, out-of-pocket costs for the patient, working incapacity, educational and employment status, invalidity and need of care, quality of life using EQ-5D and visual analogue scale (VAS) have been recorded by patient questionnaires every 3 months. All the variables of the data relevant to the study at hand are presented in Table 1.

**Table 1:** Definition of characteristics used, Germany (2009–2012)

Employment status	Employed
	Unemployed
	Houseman
	Apprentice, student, intern, military or civilian service
	Early retired
Socio-demographic factors $(i)$	
Patient age (in years)	18–29
	30–39
	40–49
	50–59
Education level	College degree
	Apprenticeship
	None of both
Health-related factors $(j)$	
Frailty (problems with daily activities)	No
	Yes
Diagnosis pre-cART (before 1996)	No
	Yes
HIV stage according to CDC classification	A (asymptomatic)
	B (symptomatic)
	C (AIDS-defining diseases)
HCV-PCR-positive hepatitis C co-infection	No
	Yes
Concomitant psychiatric disease	No
	Yes

The item frailty was assessed using item C of EQ-5D (problems with general activities, e.g. job, studies, homework, family or leisure activities). Clinical staging of HIV disease is performed in accordance to the CDC classification for HIV infection (CDC 1992). For a more comprehensive understanding, two explanatory notes are worth mentioning: By definition of the CDC classification, the staging reflects the most advanced stage of disease and does not allow a reassignment to a less advanced stage, not even in cases with a complete and stable recovery from immunodeficiency and AIDS-defining diseases under cART. More recent revisions of the CDC classification were not used in this analysis as they give less accurate information about the particular clinical stage although they are favourably for epidemiological surveillance.

**Fig. 1:**
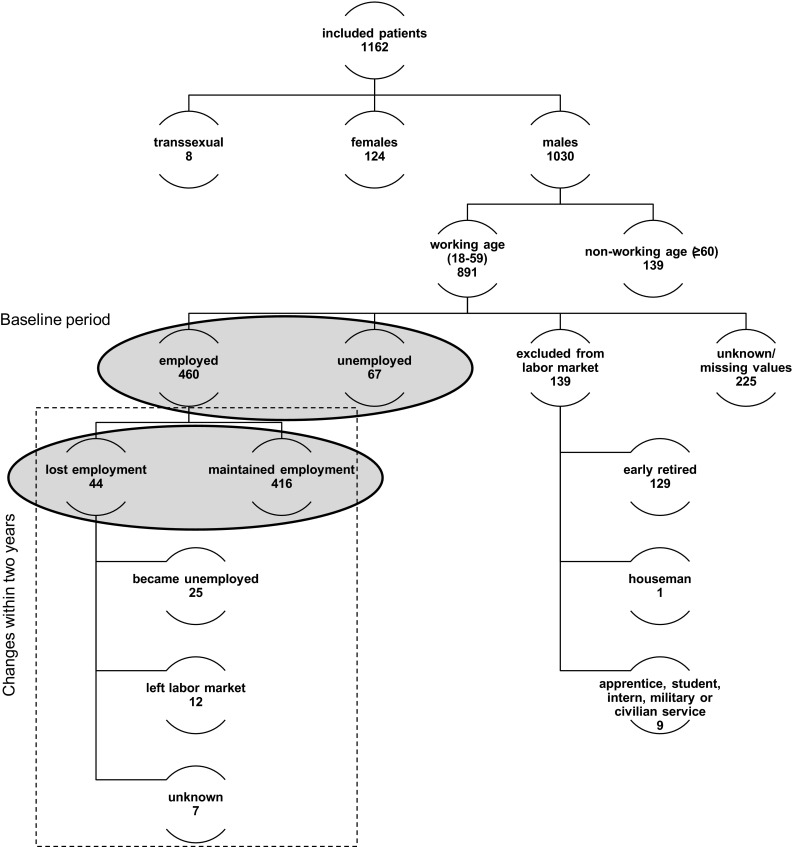
Sample description, Germany (2009–2012)

As shown in Fig. 1, the present analysis restricts the sample to male PLWHA in working age (18 to 60 years). 124 female and 8 transsexual PLWHA are excluded since they form a very small group among the PLWHA in general and they entail special socio-demographic and labour market characteristics and would thus confound the conclusions we derive for men even if controlled for gender. Similarly, regarding different policy measures frequently occurring on the German labour market, we defined working age as an age of less than 60 years. By dropping observations with missing or not useable values for the observed characteristics (225) as well as disregarding PLWHA who had already left the labour market (e.g., early retired or housemen) or who have not yet entered (e.g. students or apprentices), we generate the final sample of 527 PLWHA. All these are assumed to be available in the labour market at the beginning of the study (either working or searching for a job).

Correlations between employment status and socio-demographic as well as health-related factors were analysed from two perspectives. The first part concentrates on the baseline period, identifying differences between the employed (460) and unemployed (67) PLWHA. The second part also covers the changes of employment status within the sample of employed PLWHA at baseline. Therefore, we distinguish between PLWHA staying employed (416) and those losing employment (44) at some point in time during the observational period. Nevertheless, the latter analysis is also based on the baseline information (cross section) but uses information from the latest wave we can observe the individual. To check for robustness we drop those 33 employed that leave the sample with a lower duration. It turns out that sample attrition does not play a role. Only three out of the 44 individuals lose their job and leave before the study ends. We keep them in our sample.

The sample used in this study shows a relative low unemployment rate (12.7 %) compared to the total population (9.1 % in 2009), which is on average healthier. Since the individuals are a representative subset of people living with HIV/AIDS but—as in most medical studies—have not been selected with respect to their job status, our dataset may overrepresent people with jobs who are more willing to participate. Thus, our results may underestimate the effect of HIV/AIDS on unemployment or job loss. The estimated effects of the socio-economic and health characteristics may be much severe for the average person living with HIV/AIDS.

Having said that, first, we provide descriptive evidence for differences between the two respective groups in both samples. Since both outcome variables are binary (0 = employed, 1 = unemployed/0 = maintenance of employment, 1 = employment loss), we, then, run logistic regressions to identify statistical significant correlations with unemployment or employment loss in PLWHA. The results are stated as odds ratios (OR). This analysis therefore applies the following multivariate logistic regression models:

1 $${\text{OR}}_{\text{unemployed}} = e^{{\beta_{0} + \mathop \sum \limits_{i = 1}^{I = 2} \beta_{i} {\text{socio}}\; - \;{\text{demographic factor}}_{i} + \mathop \sum \limits_{j = 1}^{J = 4} \gamma_{j} {\text{anamnestic factor}}_{j} + \varepsilon }}$$

2 $${\text{OR}}_{\text{employment loss}} = e^{{\beta_{0} + \mathop \sum \limits_{i = 1}^{I = 2} \beta_{i} {\text{socio}}\; - \;{\text{demographic factor}}_{i} + \mathop \sum \limits_{j = 1}^{J = 4} \gamma_{j} {\text{anamnestic factor}}_{j} + \varepsilon }}$$

An OR greater than one shows that PLWHA with this characteristic face a higher chance of unemployment or job loss. All statistical analyses were performed using SPSS, Chicago and STATA, College Station. The *p* value <0.05 (two sided) is considered as being statistically significant.

## Results

### Baseline characteristics of the sample

As discussed above, the present study is restricted to 527 male, working aged PLWHA who were either employed (460) or unemployed (67) at baseline (Fig. 1). On average, those included PLWHA were first diagnosed with an HIV infection 8 years ago (range 2 days to 30 years) at an age of 34 years (range 11 to 56 years).

At baseline, a minority of the observed PLWHA is younger than 40 years (27.8 %), most of them belong to the group of 40 to 49 years of age (48.3 %). This corresponds well to the general PLWHA population in Germany (RKI 2013a, b). A college degree or an apprenticeship is documented in 16.5 and 48.2 % of the patients, respectively, whereas 35.4 % had none of both. In this respect, our sample population differs slightly from the general German population (58.6 % with an apprenticeship, 14 % with university degree and 26.7 % with none of both) (DESTATIS 2014).

16.3 % of the study population reported problems to manage daily activities (defined as frailty). The substantial majority (72.6 %) have been diagnosed with HIV/AIDS after 1996 and therefore during the cART treatment era. Displaying the disease progression, the three different categories of the Centers for Disease Control and Prevention (CDC) Classification are similarly represented within the study population: 31.6 % report CDC stage A, 39.6 % report stage B, and 28.8 % report the most severe stage C. Only 4.6 % of the PLWHA suffer from an additional HCV co-infection. 19.7 % of the patients report a concomitant psychiatric disease.

### Analysis by employment status

In the following, we distinguish the two samples highlighted in Fig. 1. First, we will discuss differences between employed and unemployed PLWHA at the beginning of the study period before we will look at the probability to lose a job within the time period of observation.

### Employed vs. non-employed patients at baseline

**Fig. 2:**
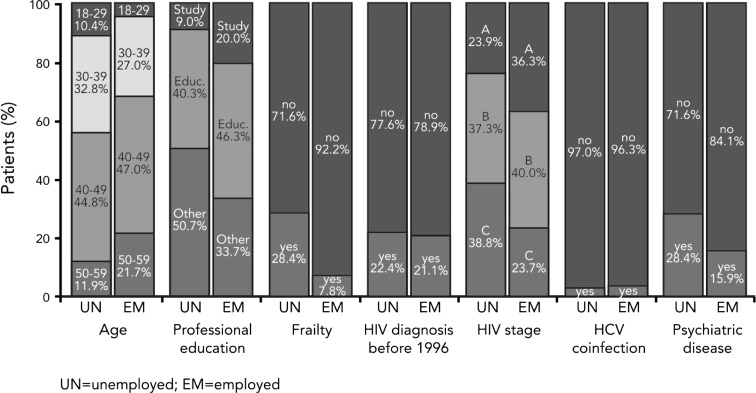
Baseline characteristics of employed (*n* = 460) vs. unemployed (*n* = 67) people living with HIV/AIDS, Germany (2009–2012)

Comparing the average characteristics of employed versus unemployed PLWHA at baseline reveals differences according to the employment status (Fig. 2). The descriptive statistics show that unemployed PLWHA face more issues regarding their measured (HIV stage, psychiatric diseases) and their self-assessed health (frailty). We test for pairwise differences in means by employment status and find that all means are significantly different from each other on a 5 % significance level except of HCV co-infection, duration since diagnosis and diagnosis before 1996. 28.4 % of unemployed patients have reported frailty compared to 7.8 % of employed patients. Unemployed patients have a more progressed HIV disease (AIDS) than employed patients: HIV stage C is found in 38.8 % of the unemployed and in 23.7 % of the employed PLWHA. HCV co-infection is found in 3.0 % of unemployed and 3.7 % of employed PLWHA, which implies that the difference between the two groups is small for this health factor. Unemployed patients suffer more frequently from concomitant psychiatric diseases (28.4 %) than employed patients (15.9 %).

Interestingly, not only health related but also socio-demographic factors differ between the two groups. First, unemployed patients are more frequent in the two younger age categories and less frequent in the two older age categories. Second, 66.3 % of the employed compared to 49.3 % of the unemployed PLWHA have either a university degree or a completed apprenticeship.

**Fig. 3:**
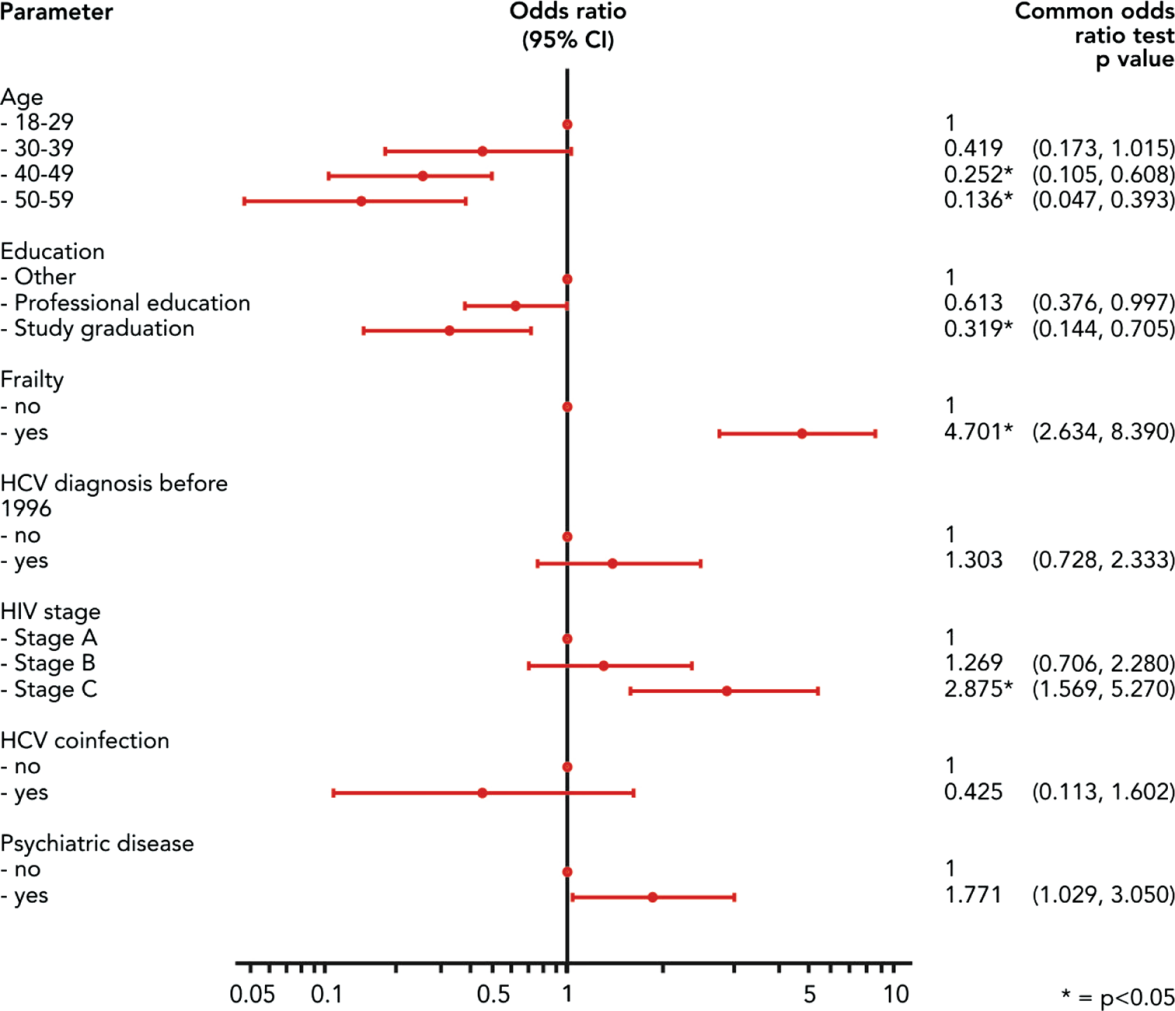
Logistic regression, multidimensional (employment vs. unemployment at baseline), Germany (2009–2012). Unemployed participants at baseline (1) compared with those who are employed at baseline (0)

By means of a logistic regression we identified factors, which are significantly correlated with the risk of unemployment in a multivariate framework (Fig. 3). The regression analysis shows five categories to be statistically significant (*p* < 0.05): Frailty is clearly an important factor for employment and shows not only a significant but also very high OR. Similarly, the HIV stage C hinders PLWHA to work, while HIV stage B is not distinguishable from stage A. The coefficients on HCV co-infections, a diagnosis before 1996 or psychiatric diseases show the expected signs but are not statistically different from zero on a 5 percent level.

However, and more interestingly, the coefficients on the two older age groups confirm our earlier observations that unemployment is more frequent in younger PLWHA below 40 years. This may be explained by more work experience, long-term contracts and a higher stock of human capital of older employees. These characteristics may make firms reconsider losing those valuable employees. Furthermore, a higher average age of the employed but a similar rate of diagnosed HIV before 1996 may also imply that the illness had been diagnosed later in life where the PLWHA had been settled in the labour market already before the diagnosis. Sample selection may be also an issue if the earlier diagnosed employed have already died due to their illness. The regression results confirm that a high educational level, specifically a university degree, significantly correlates with a lower risk of unemployment also among PLWHA. The line of reasoning may follow the above discussion about differences across age groups. Additionally, health issues may play a smaller role in higher paid jobs, being often less physically demanding.

### Loss of employment during the study period

**Fig. 4:**
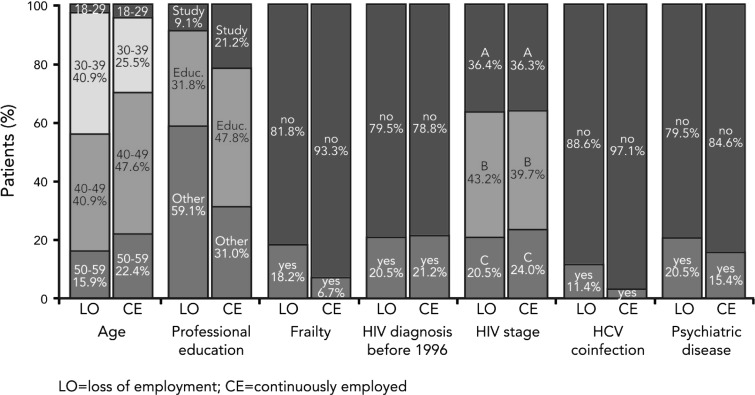
Baseline characteristics of patients with loss of employment during observation (*n* = 44) vs. patients being continuously employed (*n* = 416), Germany (2009–2012)

The data show several differences between patients being employed throughout the complete observational period vs. patients becoming non-employed during the observational period (Fig. 4). Patients losing their employment during the 2-year period report a higher degree of frailty at baseline compared to those who stayed employed (18.2 vs. 6.7 %). The socio-demographic and health factors discussed and used are defined at baseline and therefore do not vary over time. We use information on the employment status up to 2 years after the first participation to distinguish the two groups (employed throughout versus job loss) at baseline. They also suffer more often from an HCV co-infection (11.4 vs. 2.9 %) as well as from a concomitant psychiatric disease (20.5 vs. 15.4 %).

Similar to the static perspective, the younger PLWHA were worse off at the labour market. This is shown by the fact that 43.2 % of the patients becoming non-employed were younger than 40 years old. In contrast, the proportion of people in the same age group maintaining their job is 30.1 %. A lower educational level also appears to be related to higher probability of an employment loss. Therefore, a job loss seems to correlate with a bad health condition on the one hand and with socio-demographic aspects of the patient which are not directly connected to the HIV/AIDS infection on the other hand.

**Fig. 5:**
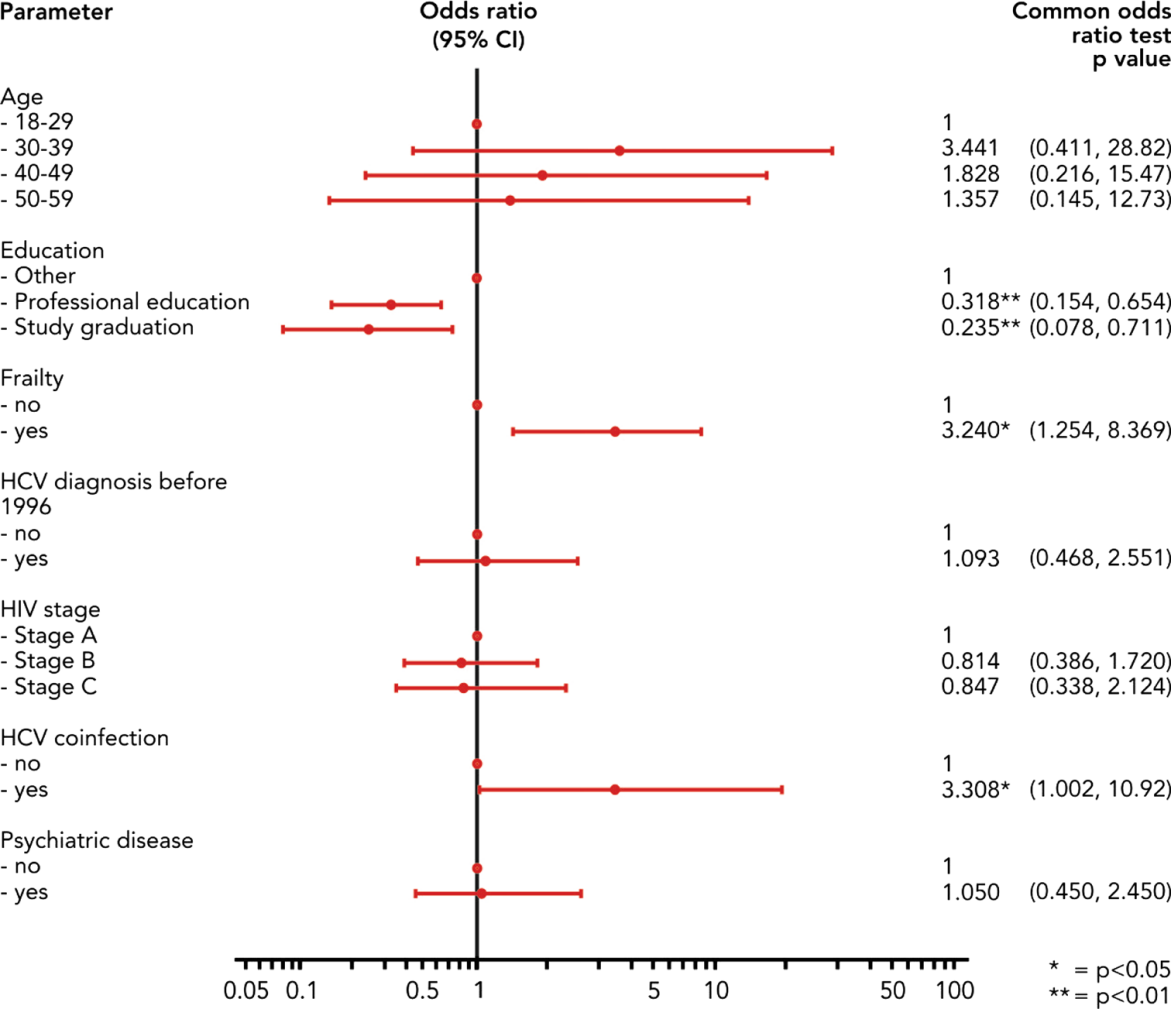
Logistic regression, multidimensional (loss of employment vs. continuous employment), Germany (2009–2012). Participants with employment loss (1) compared with those with maintenance of employment (0)

By means of a multivariate logistic regression, we were able to confirm these observations and identified four factors statistically significantly correlating with job loss (Fig. 5). PLWHA, which had stated themselves to have problems managing their daily life at baseline and therefore had been classified as frailty, were more likely to switch to non-employment during the observational period. It seems obvious that someone who struggles doing household chores, family or leisure activities and especially who struggles at work faces a higher risk of losing his job—regardless whether that loss is due to his own decision or if he was fired. In addition to the self-assessed health, the regression analysis shows that the odds of losing employment for a PLWHA with an HCV co-infection are 3.308 times larger than the odds for a PLWHA without it.

The most significant factors correlated with employment loss were the two variables concerning the education (*p* < 0.01). The higher the educational level, the less likely is the loss of employment. The younger PLWHA did not show a significantly higher probability to lose a job within this more homogenous group of people with a job at the beginning of the study period. However, we again show that the educational level also here makes a huge difference with respect to the active labour market participation of chronical ill.

## Discussion

Male PLWHA in Germany still differ from the general German population in terms of their labour market participation. In our sample, the unemployment rate at baseline lies at 12.7 %, which is almost 40 %higher than the overall German unemployment rate of 9.1 % (DESTATIS 2013). Both, socio-demographic and disease-specific characteristics of PLWHA in Germany are related to their employment status. Specifically, within the socio-demographic factors, we find age below 40 years and a poor educational level to be significantly correlated with the probability to be unemployed. Within the disease-specific factors, disease severity, concomitant psychiatric diseases, and frailty are associated with unemployment. When it comes to job loss, we identify a HCV co-infection as an additional significant factor.

Our results are well in line with the previous literature that suggests a negative relationship between chronic illnesses and employment (Alavinia and Burdorf 2008), especially among PLWHA (Worthington et al. 2012). On the other hand, the observed unemployment rates within PLWHA in Germany are much lower compared with the rates of 45–65 % reported by Dray-Spira et al. (2006, 2007a) or 40 % unemployed male PLWHA reported by Rabkin et al. (2004). The lower unemployment rates in our data might be related to national particularities, such as an overall lower unemployment rate compared to France (Dray-Spira et al. 2006, 2007a, b) or other low-income countries (Rabkin et al. 2004) as well as the selection of patients with a long-term cART treatment in our cohort. In addition, the discrepancies may reflect recent developments in prevention of disease progression, an improvement in HIV-related workplace discrimination (Dray-Spira et al. 2008), and the increasing availability of better treatment options with fewer side effects that allows patients to remain in the work place.

Identifying factors associated with unemployment, our results show that unemployment is more frequent in younger patients and corresponds with poorer education, frailty and progressing HIV stage. Interestingly, we can show that even though younger PLWHA are more frequently unemployed, they do not suffer significantly more of job losses. This may indicate that young PLWHA are less successful to find a job, but once in employment, they do not face a higher risk of job loss. This can partly be attributed to the strict German labour market that constitutes high hurdles for dismissal of regular workers. The inverse effect of education on both unemployment and job loss was expected and corresponds with the results of previous studies among the German general population (Lauer 2003). However, unlike our results, within the general population the risk of losing a job seems to be higher for those above the age of 55 compared to those between 25 and 50 years, given tenure, education and past unemployment, but not controlling for health status (Wichert and Wilke 2012).

Reported frailty does not only correspond with unemployment, it also turns out to be statistically significantly correlated with job loss. Frailty is recently recognised as a common consequence of both HIV infection and cART (Deeks 2011; Erlandson et al. 2013). Actual guidelines for the treatment of HIV-infected individuals recommend antiretroviral regimens consisting mainly of two nucleoside reverse transcriptase inhibitors (NRTI) combined with either a non-nucleoside reverse transcriptase inhibitor (NNRTI) or a boosted protease inhibitor (PI/r) or more recently an integrase strand transfer inhibitor (INSTI), respectively (DAIG 2014). Premature ageing as a potential toxic effect of cART has been primarily explained by inhibition of intracellular telomerase activity by several antiretroviral NRTI (Leeansyah et al. 2013). As NRTI are elements of virtual all treatment regimens of cART and are currently regarded as the backbone of any HIV treatment, almost all individuals in our study received NRTI-based cART. Hence, we are not able to control for such a potential effect of NRTI intake on frailty. However, recent studies investigated NRTI-sparing regimens in comparison to NRTI-based regimen for the initiation cART in PLWHA (Raffi et al. 2014). Future analysis of such studies might show, whether frailty will be delayed in PLWHA receiving NRTI-free treatment regimen and by that preserve their ability to work.

Progression to advanced stages of HIV disease is another important risk factor for unemployment in our study. Progressing to CDC stage C significantly increases the risk of unemployment. As the CDC classification does not allow a reclassification to a less advanced stage after successful treatment of an intercurrent HIV-associated or AIDS-defining disease, the staging does not reflect the actual degree of disease activity. This might explain, why the advanced stage (CDC-C) is associated with an increased unemployment rate but not with job loss during the 2-year observational period. Nevertheless, the prevention of disease progression by timely initiation of cART remains a major medical challenge. Most actual treatment guidelines recommend an earlier start of antiretroviral therapy as compared to the previous decade. However, despite today’s effective and available antiretroviral treatment, a high proportion of PLWHA are reported as late presenters, whose first consultation with a physician takes place when the disease has already progressed to severe stages. Accordingly, the number of AIDS cases has increased in some European countries (Likatavicius and Van de Laar 2012). A better screening of high-risk individuals to prevent late presentation might serve as a potential solution.

Chronic HCV co-infection did not appear as a significant risk factor for unemployment but for job loss. Previous research established a link between an HCV co-infection and a decreased quality of life (Tillmann et al. 2006), increased unemployment (March et al. 2007), lower productivity, and higher absenteeism (Brook et al. 2011). Moreover, HCV patients who required a liver transplantation were found to remain unable to return to the labour market (Huda et al. 2012). Our results suggest a higher risk of HCV co-infected patients to lose their job. This might be due to severe fatigue which is a typical symptom of HCV. Fatigue is even reinforced when the HCV infection is treated with an interferon-based treatment regimen (Huckans et al. 2015), which has been the standard treatment during our observation period. Only in 2014, the first interferon-free treatment options had been introduced to the market (Yau and Yoshida 2014). However, the small sample size of HIV–HCV-co-infected PLWHA leads to a call for future research in this particular field. It has also to be shown to what degree the new interferon-free treatment options do exert positive externalities on the labour market and protect patients from job loss. It would also be interesting to study whether the observed job loss is based on a termination by the employee or by the employer.

Psychiatric comorbidities also showed higher rates in unemployed patients. This variable constitutes probably both as the cause of unemployment as well as one of its effects. Many studies established an adverse effect of unemployment on psychosocial health as it leads to resignation, withdrawal, and decreased self-esteem (Weber et al. 2007).

While our study looked only at the determinants of employment, we were not able to measure job performance and productivity of HIV-infected people who are still in the labour force. There is some evidence that side effects of HIV treatment are significantly related to a lower productivity in the US (daCosta et al. 2012), and Switzerland (Sendi et al. 2004). An inverse relationship between labour productivity and HIV status is also found in emerging markets such as Kenya (Larson et al. 2013). This means that our approach underestimates the costs HIV is placing on the labour market. We also do not consider long-term costs of unemployment. As McLeod et al. (2012) were able to show, unemployment is associated with a higher mortality for low- and medium-skilled workers in the US und for high-skilled workers in Germany. One of the limitations of our “piggy back” study is that it is limited to male patients. We also cannot preclude a selection bias in our sample as some difficult-to-treat patients might not have made it into the study. This would explain the relatively low unemployment rate in our sample.

### Conclusions

Both socio-demographic and disease-specific characteristics of PLWHA in Germany are related to their employment status. This means that a timely initiated HIV treatment is an important cornerstone to prevent unemployment. The same holds for the management of comorbidities such as HCV or psychiatric diseases. Taking a long-term view, potential new treatment options in HIV that reduce frailty and disease progression as well as better education would not only be beneficial for the individual patient but also for the society as a whole.

## References

[CR1] Alavinia SM, Burdorf A (2008). Unemployment and retirement and ill-health: a cross-sectional analysis across European countries. Int Arch Occup Environ Health.

[CR2] Anandan N, Braveman B, Kielhofner G (2006). Impairments and perceived competence in persons living with HIV/AIDS. Work.

[CR3] Brook RA, Kleinman NL, Su J (2011). Absenteeism and productivity among employees being treated for hepatitis C. Am J Manag Care.

[CR4] Centers for Disease Control and Prevention [CDC] (1992) 1993 revised classification system for HIV infection and expanded surveillance case definition for AIDS among adolescents and adults. MMWR 41 (No. RR-17) 1361652

[CR5] Chen YQ, Masse B, Wang L (2012). Statistical considerations for the HPTN 052 Study to evaluate the effectiveness of early versus delayed antiretroviral strategies to prevent the sexual transmission of HIV-1 in serodiscordant couples. Contemp Clin Trials.

[CR6] daCosta D, Bonaventura M, Gupta S (2012). The association of HIV/AIDS treatment side effects with health status, work productivity, and resource use. AIDS Care.

[CR7] Deeks SG (2011). HIV infection, inflammation, immunosenescence, and aging. Annu Rev Med.

[CR8] DESTATIS (2013) Bevölkerung und Erwerbstätigkeit Stand und Entwicklung der Erwerbstätigkeit in Deutschland. https://www.destatis.de/DE/Publikationen/Thematisch/Arbeitsmarkt/Erwerbstaetige/StandEntwicklungErwerbstaetigkeit2010411127004.pdf?__blob=publicationFile. Accessed 24 Sept 2015

[CR9] DESTATIS (2014) Tabelle zum Bildungsstand 2010–2012. https://www.destatis.de/DE/ZahlenFakten/GesellschaftStaat/BildungForschungKultur/Bildungsstand/Tabellen/Bildungsabschluss.html. Accessed 24 Sept 2015

[CR10] Deutsche AIDS-Gesellschaft (DAIG) (2014) Deutsch-Österreichische Leitlinien zur antiretroviralen Therapie der HIV–Infektion 2014. http://www.daignet.de/site-content/hiv-therapie/leitlinien-1/Deutsch_Osterreichische%20Leitlinien%20zur%20antiretroviralen%20Therapie%20der%20HIV_Infektion.pdf. Accessed 24 Sept 2015

[CR11] Dray-Spira R, Persoz A, Boufassa F (2006). Employment loss following HIV infection in the era of highly active antiretroviral therapies. Eur J Pub Health.

[CR12] Dray-Spira R, Lert F, the VESPA Study Group (2007). Living and working with HIV in France in 2003: results from the ANRS-EN12-VESPA Study. AIDS.

[CR13] Dray-Spira R, Gueguen A, Ravaud J-F (2007). Socioeconomic differences in the impact of HIV infection on workforce participation in France in the era of highly active antiretroviral therapy. Am J Public Health.

[CR14] Dray-Spira R, Gueguen A, Lert F (2008). Disease severity, self-reported experience of workplace discrimination and employment loss during the course of chronic HIV disease: differences according to gender and education. Occup Environ Med.

[CR15] Erlandson KM, Allshouse AA, Jankowski CM (2013). Association of functional impairment with inflammation and immune activation in HIV type 1-infected adults receiving effective antiretroviral therapy. J Infect Dis.

[CR16] Goldman DP, Bao Y (2004). Effective HIV treatment and the employment of HIV+ adults. Health Serv Res.

[CR17] Huckans M, Fuller B, Wheaton V (2015). A longitudinal study evaluating the effects of interferon-alpha therapy on cognitive and psychiatric function in adults with chronic hepatitis C. J Psychosom Res.

[CR18] Huda A, Newcomer R, Harrington C (2012). High rate of unemployment after liver transplantation: analysis of the United Network for Organ Sharing database. Liver Transpl.

[CR19] Krebs T, Scheffel M (2013). Macroeconomic evaluation of labor market reform in Germany. IMF Econ Rev.

[CR20] Kuhlmann A, Mittendorf T, Hower M (2015). Krankheitskosten von HIV-Patienten unter antiretroviraler Therapie in Deutschland—Ergebnisse einer 48-Wochen-Interimsanalyse im Rahmen der prospektiven multizentrischen Kohortenstudie „CORSAR“. Gesundheitswesen.

[CR21] Larson BA, Fox MP, Bii M (2013). Antiretroviral therapy, labour productivity, and sex: a longitudinal cohort study of tea pluckers in Kenya. AIDS.

[CR22] Lauer C (2003). Family background, cohort and education: a French-German comparison based on a multivariate ordered probit model of educational attainment. Labour Econ.

[CR23] Leeansyah E, Cameron PU, Solomon A (2013). Inhibition of telomerase activity by human immunodeficiency virus (HIV) nucleos(t)ide reverse transcriptase inhibitors: a potential factor contributing to HIV-associated accelerated aging. J Infect Dis.

[CR24] Likatavicius G, Van de Laar M (2012). HIV and AIDS in the European Union, 2011. Euro Surveill.

[CR25] March JC, Oviedo-Joekes E, Romero M (2007). Factors associated with reported hepatitis C and HIV among injecting drug users in ten European cities. Enferm Infecc Microbiol Clin.

[CR26] McLeod CB, Lavis JN, MacNab YC (2012). Unemployment and mortality: a comparative study of Germany and the United States. Am J Public Health.

[CR27] Mühlbacher AC, Stoll M, Mahlich J (2013). Patient preferences for HIV/AIDS therapy—a discrete choice experiment. Health Econ Rev.

[CR28] Pichenot M, Deuffic-Burban S, Cuzin L (2012). Efficacy of new antiretroviral drugs in treatment-experienced HIV-infected patients: a systematic review and meta-analysis of recent randomized controlled trials. HIV Med.

[CR29] Rabkin JG, McElhiney M, Ferrando SJ (2004). Predictors of employment of men with HIV/AIDS: a longitudinal study. Psychosom Med.

[CR30] Raffi F, Babiker AG, Richert L (2014). Ritonavir-boosted darunavir combined with raltegravir or tenofovir–emtricitabine in antiretroviral-naive adults infected with HIV-1: 96-week results from the NEAT001/ANRS143 randomised non-inferiority trial. Lancet.

[CR31] Richardson L, Wood E, Li K (2010). Factors associated with employment among a cohort of injection drug users. Drug Alcohol Rev.

[CR32] Robert Koch Insitut (RKI) (2014). Analysen zur HIV-Inzidenz und -Prävalenzschätzung 2013. Epidemiol Bull.

[CR33] Robert Koch Institut (RKI) (2013) Epidemiologie 2013, Berlin 2013. http://www.rki.de/DE/Content/InfAZ/H/HIVAIDS/Epidemiologie/Daten_und_Berichte/EckdatenDeutschland.pdf?__blob=publicationFile. Accessed 24 Sept 2015

[CR34] Robert-Koch-Institut (RKI) (2013). Weiterführende Analysen zur HIV-Inzidenz- und Prävalenzschätzung 2012. Epidemiol Bull.

[CR35] Sendi P, Schellenberg F, Ungsedhapand C (2004). Productivity costs and determinants of productivity in HIV-infected patients. Clin Ther.

[CR36] Tillmann HL, Kaiser T, Claes C (2006). Differential influence of different hepatitis viruses on quality of life in HIV positive patients. Eur J Med Res.

[CR37] Weber A, Hörmann G, Heipertz W (2007) Unemployment and Health—a public health perspective. Dtsch Arztebl 104(43):A 2957–2962

[CR38] Wichert L, Wilke R (2012). Which factors safeguard employment? An analysis with misclassified German register data. J R Stat Soc Ser A Stat Soc.

[CR39] Worthington C, O’Brien K, Zack E (2012). Enhancing labour force participation for people living with HIV: a multi-perspective summary of the research evidence. AIDS Behav.

[CR40] Yau AH, Yoshida EM (2014). Hepatitis C drugs: the end of the pegylated interferon era and the emergence of all-oral interferon-free antiviral regimens: a concise review. Can J Gastroenterol Hepatol.

